# Supplementary material for: Spontaneous cortical activity transiently organises into frequency specific phase-coupling networks
Source: Nat Commun. 2018 Jul 30;9:2987. doi: 10.1038/s41467-018-05316-z (PMC6065434; doi:10.1038/s41467-018-05316-z)
Supplement: Supplementary file 1 — Supplementary Information [file 41467_2018_5316_MOESM1_ESM.pdf]

Spontaneous cortical activity transiently organises into frequency specific phase-coupling networks

Vidaurre et al.

Supplementary Discussion

Our approach identifies brain states characterized by distinct power and phase-coupling. Related to the question of how much phase and coherence contribute to state switching (see Results), there is the issue of power differences biasing the estimation of phase-coupling. Here, we find that states that show increase in power, often, although not exclusively, also show increases in coherence. However, it is well known that, due to changes in the signal-to-noise ratio, increases in power can augment the estimated coherence even in the absence of an actual change in the interactions between the regions. The same phenomena can cause increases in variance to effect correlation-based measures of functional connectivity[1,2]. Therefore, some of the observed changes in coherence between states might be caused by differences in power. Notably, while we find that power and coherence are generally positively correlated, there are various aspects of phase-coupling that cannot be explained by changes in power. For example, the differences between the states are much stronger in coherence than in power (see, e.g., **Figure 3a**). Also, whereas the PCC has slightly more (low-frequency) power in the anterior higher-order cognitive state than in the other states, phase-coupling is the feature most strongly stands out from the rest (see **Figure 3c**). However, while there are examples of coherence and power not changing together, it remains possible that many of the changes in coherence between states could be driven by changes in power.

A relevant related issue is the choice of metric used to measure phase-coupling. There are different alternatives, of which spectral coherence and phase-locking value (PLV) are two popular examples. Although PLV has been claimed to represent phase-coupling more faithfully than spectral coherence[3], in this work we found PLV to be indeed less robust at the large scale than coherence, partly because the application of our data-driven spectral decomposition (as we performed for coherence) is not directly applicable, so we had to rely on an arbitrary specification of the frequency bands. Furthermore, PLV has its own limitations, such as its dependence on filtering and the subsequent use of the Hilbert transform for instantaneous phase calculation[4] ; see also [5] for a comprehensive description of this issue. It is also worth noting that one of the main limitations of spectral coherence is stationarity, which is mitigated here by the fact that the HMM breaks, to some extent, the non-stationarity of the signal into short visits to quasi-stationary states. Further, PLV is also not automatically immune to power bias, given that phase-locking is inferred more reliably when the power (and therefore the signal-to noise ratio) is high. The advantages and disadvantages of different approaches to compute phase-coupling are however beyond the scope of this paper, as a satisfactory solution would require to properly deal with the problems of nonlinearity and non-stationarity, at the heart of the limitations of both spectral coherence and phase-locking value.

A central parameter in our approach is the number of HMM states. Here, we have chosen it to be twelve. Importantly, we do not claim this number to be closer to any biological ground-truth than, for example, eight or sixteen. Although it is possible to guide the choice of the number of states using quantitative measures like the free energy[6], or even using non-parametric approaches that automatically determine the number of states[7], different numbers of states in practice just offer different levels of detail of brain dynamics. Indeed, examining different degrees of abstraction can reveal useful insights. For example, when we ran the proposed approach with six states (see **Supplementary Figure 8**) the posterior higher-order cognitive state was fused with the first two states depicted in **Supplementary Figure 2**. This is unsurprising considering their relatively similar spectral and spatial features. In this analysis, the left and right temporal states (see **Supplementary Figure 2**), which are characterised by high asymmetry and are possibly related to language, were also merged into a single symmetric state containing the patterns of both. In summary, running the HMM with different numbers of states and combining the results in a principled way can provide a hierarchical view of the data that is hidden to other approaches. Thanks to the stochastic scheme of inference[8], HMM runs are not computationally expensive to produce, facilitating these exploratory

analyses. For a practical discussion on the choice of the other HMM parameters, refer to **Methods**. Another important aspect (and a sensible way of guiding the choice of the number of states) is state reliability, that is, how robust are the states across, for example, half-splits of the data? The question of reproducibility is discussed below.

The model specification of the HMM, through assigning state probabilities at each time point, implicitly assumes that only one state is active at each point in time. However, it is worth noting that it is still possible for network multiplexing to be realised at slower time scales through temporal correlation of the rate of occurrence of states. At the faster time-scale of HMM switching, it is important to note that any conclusion about brain network exclusivity must be made with caution and is by no means necessarily a physiologically meaningful feature of the brain. Addressing the information contained in the state time courses at multiple time scales is an important area for future investigations.

The TDE-HMM is a useful representation of the data, but is not the only possible one. For instance, a high order multivariate autoregressive model has the potential to explain very rich dynamics to similar extent, but in an alternative manner, to an HMM with a simpler observation model[9,10]. Armed with just resting data, it is not possible to disambiguate between these two different descriptions of the data. Which one is more appropriate rather depends on the question in hand. A potential reason to use the HMM over a single-state more complex model (such as a high order multivariate autoregressive model) is that it explicitly parameterises the time series through the state time courses, opening avenues to investigate, for example, the interactions between rest and task. Further, it is through the use of the HMM in this work that we have been able to successfully identify networks of spatially distinct patterns of oscillatory power and phase-coupling in specific frequency bands, in a manner that has not been achieved previously with other approaches, including the autoregressive model.

Supplementary Note 1

Slow-frequency spectral properties within fast state visits

We obtained state visits that were often well under 100ms (**Figure 5**). How can this be compatible with the slow frequencies (e.g. delta/theta bands) that characterise the states? Here, we show that this is theoretically and practically possible through simulations. We have simulated data where segments of an 8Hz theta wave are interspersed with unstructured signal. We have performed three sets of simulations. In each of them, the duration of the wave segments (which are selected at random points of the theta period) are sampled from a Poisson distribution with mean 0.025s, 0.05s, and 0.1s, respectively. The separation between segments is sampled from a Poisson distribution with mean 1s in all cases, which makes the different wave occurrences to be completely phase-independent. Small-variance Gaussian noise is added to the generated signals. We simulated 20min of data at 250Hz for each simulation, and assumed a state time course that is active only at the time of the wave segments occurrences. Hence, the duration of the wave segments corresponds to the duration of the state visits. We then used the state-wise multitaper used in Results and in [9] to assess the spectral content of the signal. **Supplementary Figure 9** shows the spectral estimation on the top, and an example of a wave segments for each mean dwell time in the bottom. In the three cases, and despite the short state visits, the state-wise multitaper is able to find the correct frequency, even when the frequency resolution is degraded somewhat as we make the wave segments (state visits) shorter, leaking power toward faster frequencies.

Supplementary Note 2

Leakage reduction and phase-locking coherence estimation

In this work, we used the method proposed in [11] in order to reduce the effect of signal leakage (volume conduction). Without this step, the estimation of phase-locking gets dominated by a pattern of artefactual local connections that is common to all states. While the approach proposed in [25] has been shown to work well in the context of MEG amplitude correlations, its application for phase-locking networks is less well established. Recently, Pascual-Marqui and colleagues[12] have challenged the aforementioned approach, particularly within the context of estimating phase-locking measures, showing that under certain conditions, artefactual connections may arise. The authors also provide an alternative approach based on the multivariate autoregressive model that may overcome these issues.

To check the approach presented in [11] in the context of phase-locking, we also applied the Pascual-Marqui's approach on our real data[12], and compared the resulting state-specific phase-locking with the estimations depicted in **Figure 2**. In **Supplementary Figure 10**, phase-locking connectivity is shown for the same four HMM states, after applying Pascual-Marqui's method. As observed, the differences between **Supplementary Figure 10** and **Figure 2** are limited, with the method proposed by Pascual-Marqui and colleagues being slightly more conservative. The main features of the HMM states are however preserved.

A related issue is whether leakage correction, which makes the signals orthogonal across the entire time series, precludes completely zero-lag (or small lag) relationships, which are central to the theory of communication through coherence[13]. Importantly, leakage correction operates at the level of the entire time series, and so only removes zero-lag correlations *on average*. This means that it is still possible to have *transient* periods of zero- or small- lag synchronisation. Focusing on the anterior and posterior higher-order cognitive networks, **Supplementary Figure 11** illustrates this point by showing the phase at which different regions have a high coherence with the PCC (using a threshold of 0.05). Each dot thus represents a region with high coherence with PCC at the indicated frequency. Colours represent large-scale cortical areas. Importantly, due to the sign ambiguity issue, it is not possible to distinguish in-phase (0) from anti-phase coherence (π). If we assume that anti-phase actually represents in-phase relationships, this figure suggests that many of the (transient) phase-locking relationships are actually close to zero-lag.

Supplementary Note 3

Reproducibility

We assessed the reproducibility of the states by randomly splitting the data into two groups of subjects (half-splits) and running five times the HMM inference separately on each half. We also ran the HMM on the full data set five times. This is intended to evaluate the reproducibility of the results both across different HMM runs and across different subjects. We obtained 12 states from each run and matched the states across runs (between the two partitions and to the full cohort run) such that the similarity between state pairs is maximal. We used Riemannian distances to quantify the dissimilarities between states (see Methods). **Supplementary Figure 12a** shows the Riemannian distances between each pair of states (within and between runs). We then performed statistical testing on the consistency between runs across halves. Conceptually, we aimed to test if each pair of matched states (one per half) reliably represents the same process. If the distance between the states within the pair is significantly lower than between any two non-matched states, the state represented by this pair is robust across runs and subjects. This is shown in **Supplementary Figure 12b** (left), where several states appear to be significantly reliable. A notable exception is the anterior higher-order cognitive state, the reason being its high similarity with state 5 (see **Supplementary Figure 12a**); that is, these two states are relatively similar and can potentially be mixed in certain runs. If, on the other hand, we test the distance of each pair of matched states against the *average* distance between any pair of states (that is, a less conservative test), the anterior higher-order cognitive state appears as highly reliable (**Supplementary Figure 12b**, right). In summary:

- Overall, there is a strong similarity across runs, both within and between half-split partitions of the data (and to the full data set).
- Within sessions, some states are relatively similar, suggesting some form of state hierarchy.

Supplementary Note 4

HMM states from surrogate data

In this paper, we have proposed a model that finds separable, spectrally-defined states from MEG data. Applied on the resting-state, we found a number of states with interpretable characteristics. Being aware that the brain complex dynamics can be equally well represented in multiple ways (see above), it is important to investigate how the HMM states might differ between those we find in surrogate data simulated from complex single-state dynamic models, and those that we find in the real data. To test this, we implemented a surrogate data generation procedure where we kept analogous 1/f dynamics and autocorrelations while breaking the state-specific dynamics by using autoregressive models. In particular, using the 42-channels data used in the rest of the paper before leakage correction, we estimated two models: (i) a multivariate autoregressive model of order 3 (MAR(3), with 42^2 x 3 parameters) which captured between channel autocorrelations, and (ii) a collection of univariate autoregressive models (one per channel) of order 21 (AR(21), with 21 x 42 parameters) which did not capture between-channels spectral characteristics, but which was able to estimate more detailed within-channel spectral features. We then sampled data from these two models, corrected for signal leakage, and applied the HMM.

Consistent with our expectations, and as discussed in previous work[9,10], the autoregressive model is indeed able to represent complex dynamics. Depending on the complexity of the autoregressive model (in particular, in the case of the MAR(3) and AR(21) models), a single (low-rank) lagged cross-correlation (as corresponds to the HMM states in our model) cannot represent the data well enough, and, therefore, several states are necessary. In other words, complex 1/f and cross-spectral dynamics can either be represented by an autoregressive model with a large number of parameters, or a set of less complex models as we use in this work. As a consequence of this, different HMM states emerge from these simulated data.

We compared the HMM decomposition obtained from the real data and the HMM decomposition obtained from these synthetic scenarios. **Supplementary Figure 13** shows the results. The states obtained from real data hold significant differences with the states obtained from the MAR(3) and the AR(21) models. As a first approach, we compared the wideband spectral maps (see Methods) between the surrogated and the real states. For this, we paired up the synthetic states with the real states such that the correlation is maximal. In the MAR(3) case, the states have relatively low correlations between real and synthetic (top left); in the AR(21) case, which captures the within-channel spectral information more faithfully, the correlation is much higher, with the interesting exception of the higher-cognitive states and the fifth state (top right). As demonstrated in the bottom-right panel, these high correlations are however trivially explained by the across-states average power profile being very similar between synthetic and real states; this was expected given the AR(21) model's high explanatory power in the spectral domain. Note that this grand-average correlation is missed in the MAR(3) model case, most likely due to leakage correction removing a large extent of the (stationary) information. Most importantly, the states obtained from the real data are much more diverse than those corresponding to either of the synthetic models, as shown in the bottom panels. This, together with the inability to identify the higher-cognitive states, suggests that the HMM states obtained here are not trivially obtained from just any data with the same spatiotemporal autocorrelations.

Supplementary Methods

Simulating data from the HMM

Each state model of the TDE-HMM corresponds to a Gaussian process[14]. In order to generate *T* time points of data from a given state, one can construct a (no. of regions x T) by (no. of regions x T) covariance matrix by rearranging the elements of the (no. of regions x time lags) by (no. of regions x time lags) state covariance matrix. In order to generate data from the TDE-HMM, we used the two higher-order states and selected a subset of regions for computational simplicity (ACC, PCC, and left and right intraparietal sulci). We then sampled 30min of data, alternating between these two states, with state visits set to last from 0.2 to 2s. We then ran the HMM inference on the simulated data. The inference, as shown in **Supplementary Figure 14**, was able to accurately recover the simulated state time course with a correlation between simulated and estimated time courses of *r*=0.98. Furthermore, when simulating from one single state, the HMM inference was able to reduce the complexity of the model by eliminating all states but one.

Supplementary Table

Index	Area	MNI Coordinates (X,Y,Z)
1	Medial PFC	(2,50,0)
2	Right Frontal Lobe	(42,34,16)
3	Right Frontal Lobe	(26,10,56)
4	Right Frontal Lobe	(18,42,40)
5	Right Frontal Lobe	(42,50,0)
6	Right Frontal Lobe	(26,50,24)
7	Right Temporal Lobe	(58,-22,8)
8	Right Temporal Lobe	(58,-46,0)
9	Right Temporal Lobe	(50,10,-24)
10	Right Visual Cortex	(10,-94,24)
11	Right Visual Cortex	(26,-94,8)
12	Right Visual Cortex	(50,-70,8)
13	Right Visual Cortex	(42,-78,-8)
14	Right Sensorimotor Cortex	(58,-6,32)
15	Right Sensorimotor Cortex	(42,-22,56)
16	Right Sensorimotor Cortex	(10,-30,72)
17	Right Parietal Lobe	(18,-70,56)
18	Right Parietal Lobe	(34,-78,40)
19	Right Parietal Lobe	(-54,-46,40)
20	Right Parietal Lobe	(50,-70,16)
21	Posterior Precuneus	(-6,-70,32)
22	Posterior Cingulate Cortex	(2,-46,24)
23	Anterior Precuneus	(2,-54,48)
24	Left Parietal Lobe	(-22,-62,56)
25	Left Parietal Lobe	(-38,-78,40)
26	Left Parietal Lobe	(58,-46,40)
27	Left Parietal Lobe	(-46,-70,16)
28	Left Sensorimotor Cortex	(-54,-6,32)
29	Left Sensorimotor Cortex	(-46,-22,56)
30	Left Sensorimotor Cortex	(-6,-30,72)
31	Left Visual Cortex	(-14,-94,24)
32	Left Visual Cortex	(-22,-94,8)
33	Left Visual Cortex	(-46,-70,8)
34	Left Visual Cortex	(-38,-86,0)
35	Left Temporal Lobe	(-62,-22,8)
36	Left Temporal Lobe	(-62,-46,0)
37	Left Temporal Lobe	(-46,10,-24)
38	Left Frontal Lobe	(-46,34,16)
39	Left Frontal Lobe	(-22,10,56)
40	Left Frontal Lobe	(-14,42,48)
41	Left Frontal Lobe	(-38,50,0)
42	Left Frontal Lobe	(-22,58,16)

Supplementary Table. List of regions used in the analysis, with their MNI coordinates. Numerical indexes correspond to those shown in **Figure 2**, **Figure 4**, and **Supplementary Figure 4**.

Supplementary Figures

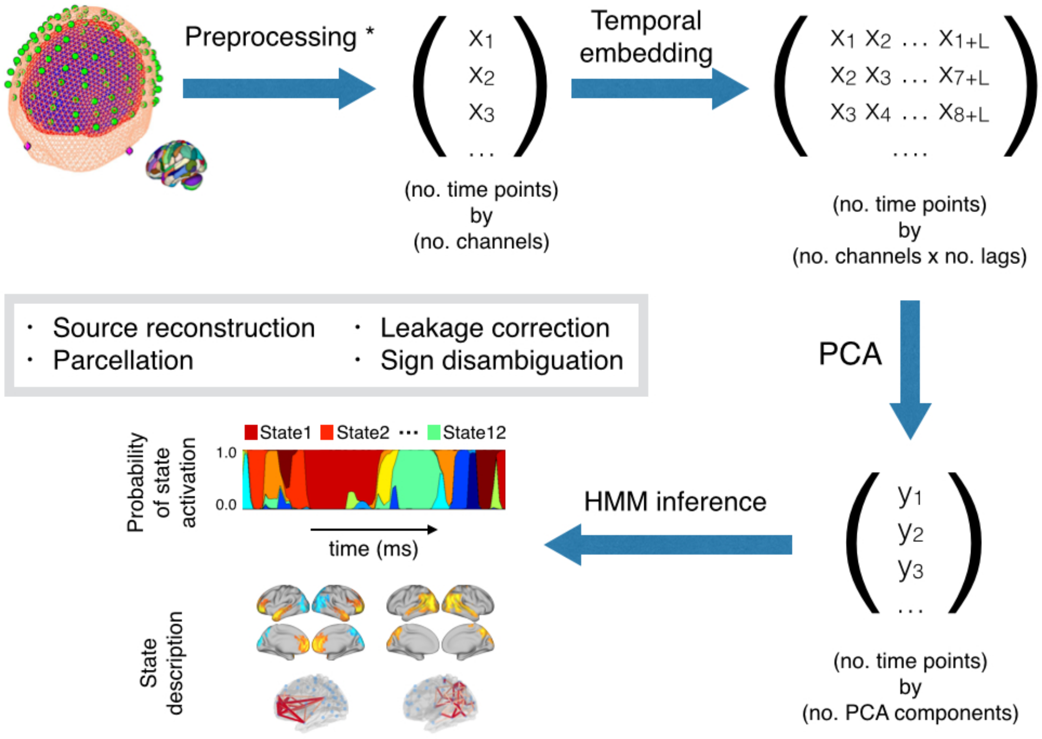

Supplementary Figure 1. Schematic overview of the proposed method. After preprocessing (source reconstruction, parcellation, leakage correction and sign disambiguation – see Methods), the data channels *X* are temporally-embedded (using *L* lags), PCA is applied for dimensionality reduction (producing PCA components *Y*), and HMM inference is then used to find the state time courses and the state parameters.

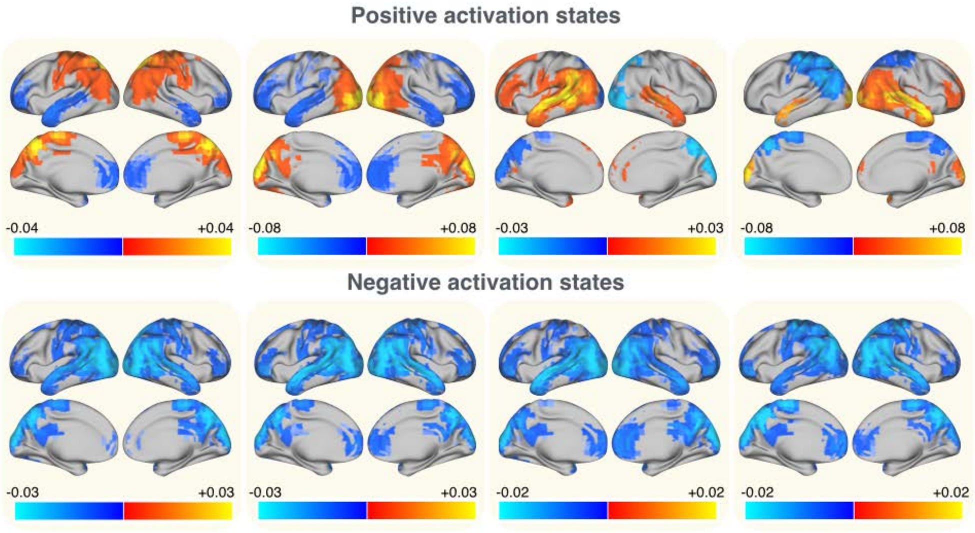

Supplementary Figure 2. **The remaining eight states of the HMM model**. Four of these (in the bottom) correspond to depressed (with regard to average) power and connectivity.

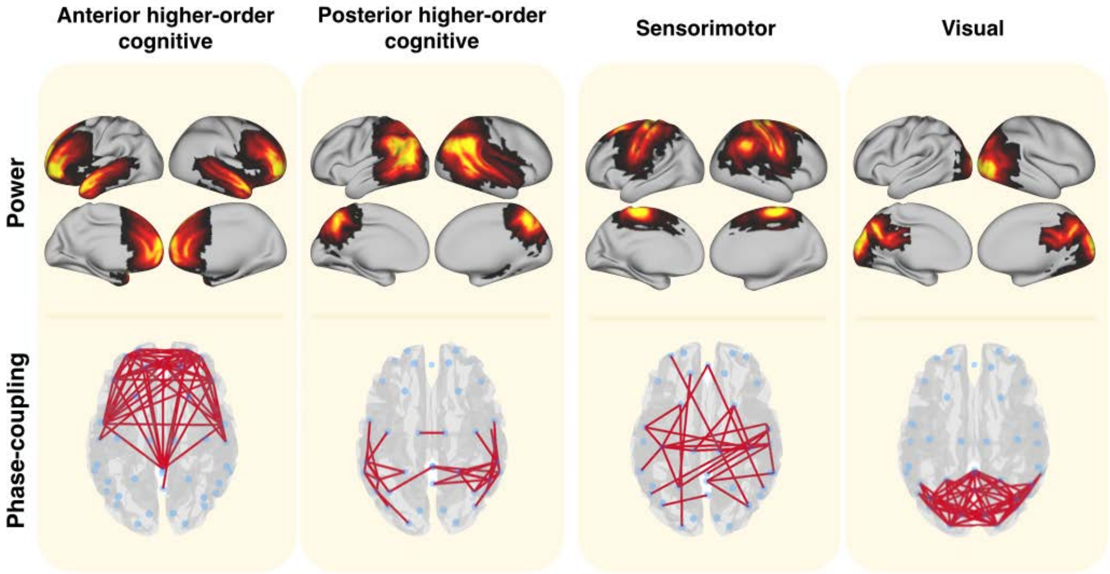

Supplementary Figure 3. **Statistically-significant wideband activations and functional connections, for the four states depicted in Figure 2**. Tested hypothesis refer to activations or connections to be higher for these states than for the rest of the states. The level of statistical significance was set to 0.01.

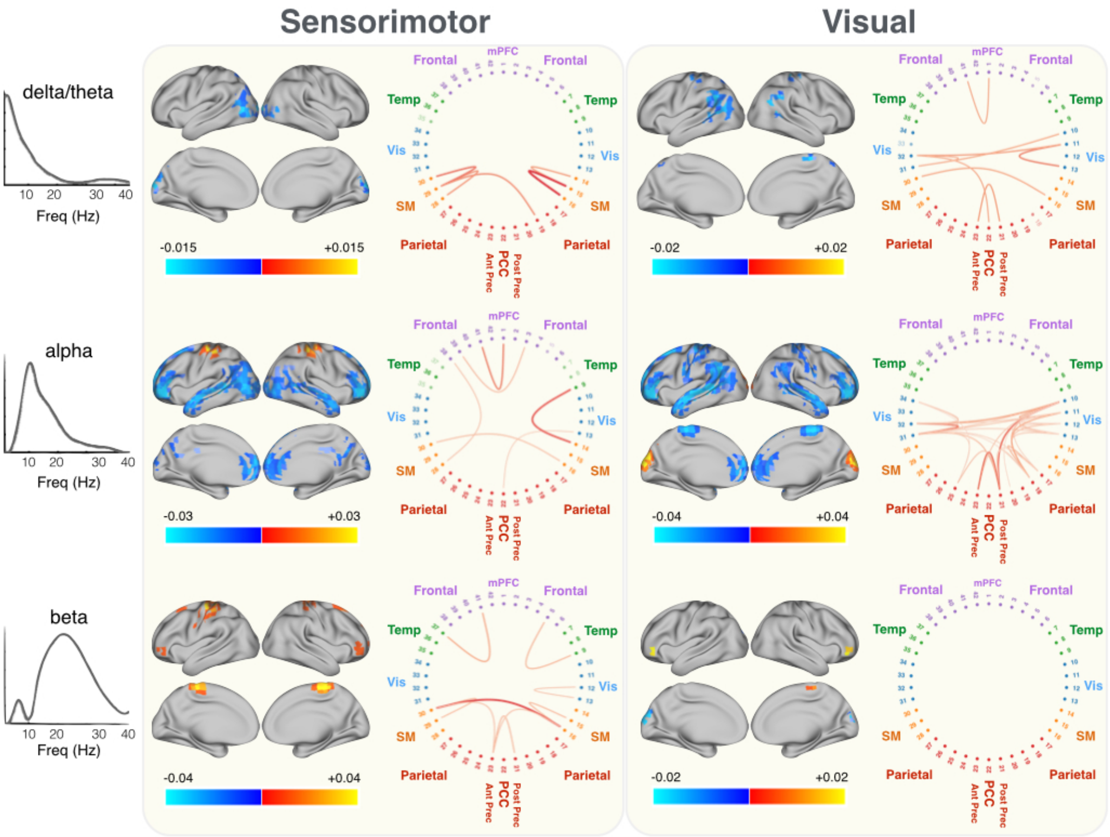

Supplementary Figure 4. **Frequency-specific power and phase-locking connectivity for the visual and motor states**. This is shown for the three data-driven estimated frequency modes (see Methods). With regard to connectivity, only the connections with highest absolute value are shown (see Methods).

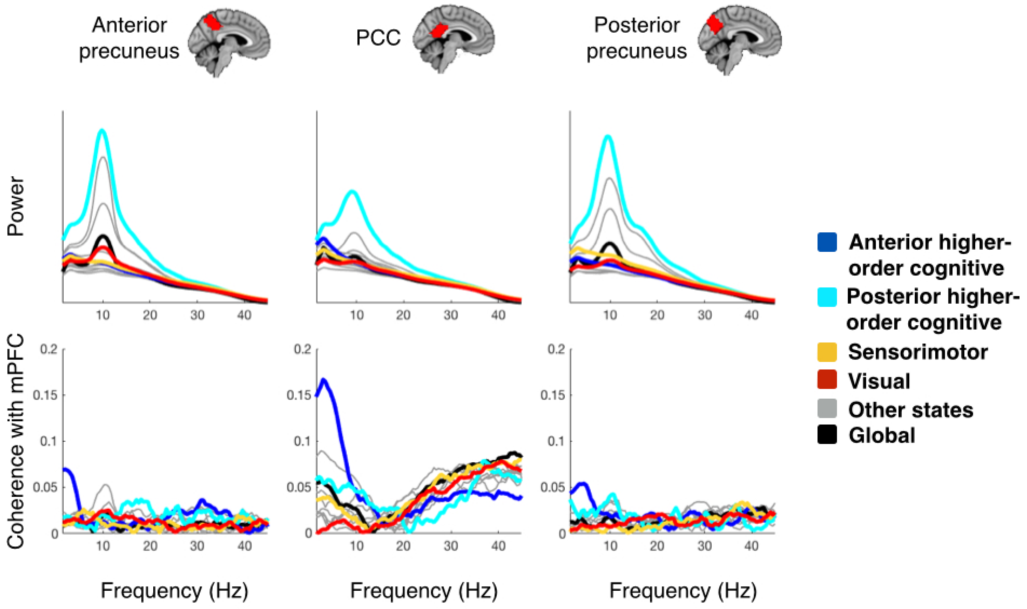

Supplementary Figure 5. **The spectral profile of the three regions is distinct when considered state by state**. The top three panels represent power as a function of frequency for the PCC and the two precuneus regions, separated by state and including the global power. The bottom panels reflect connectivity with the mPFC region.

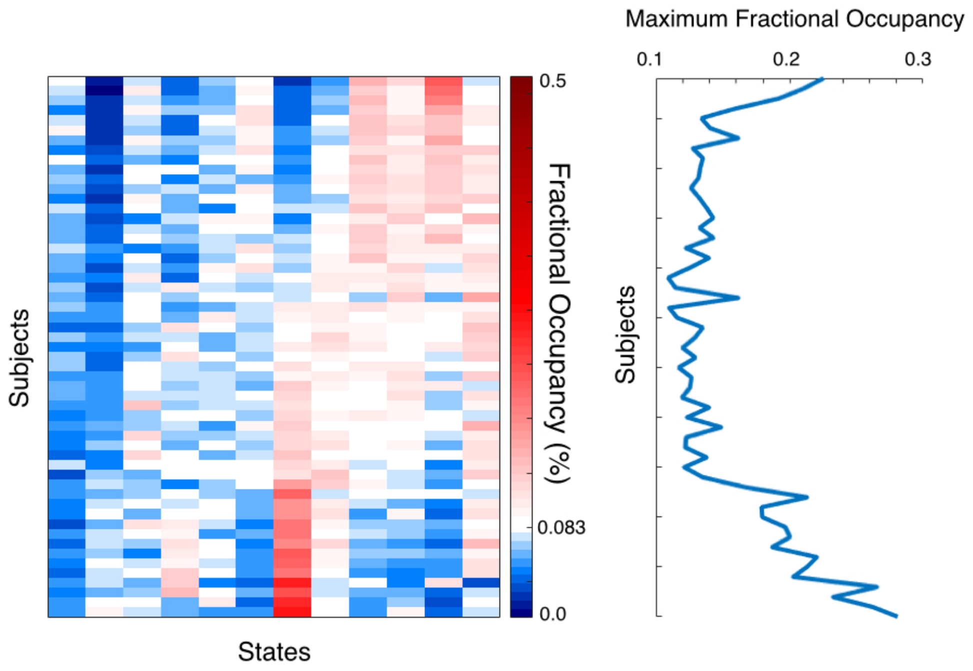

Supplementary Figure 6. **The state distribution is not homogenous over subjects, with some subjects having a higher representation of some states than others**. On the right, the maximum fractional occupancy (per subject) indicates that all subjects are explained by a mixture of states, and that no single state dominates an entire subject.

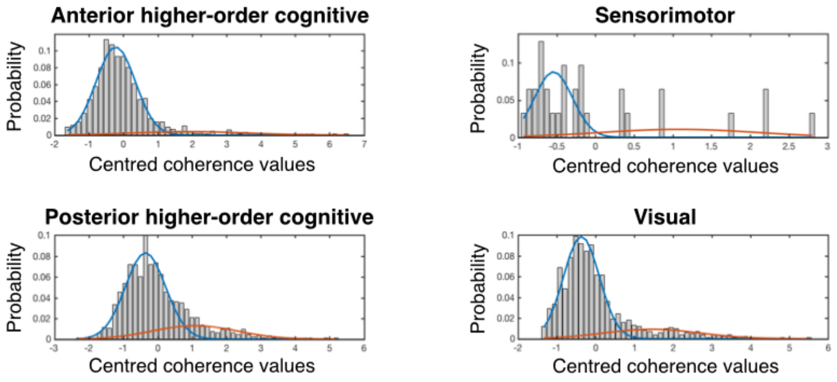

Supplementary Figure 7. **Histogram and fitted Gaussian distributions used to select the relevant connections in Figure 2**. These are chosen by fitting a mixture of two Gaussian distributions to the state's distribution of connections, and selecting the ones that belong to the Gaussian distribution with the larger mean.

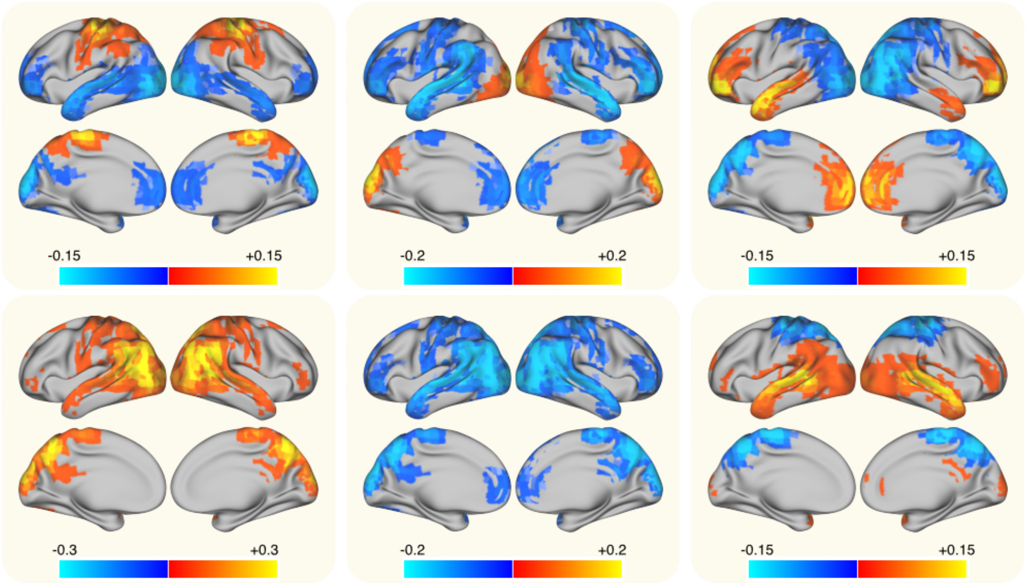

Supplementary Figure 8. **Spatial power maps for an HMM run with 6 states**. Some states from the original 12 state analysis are now fused into fewer states.

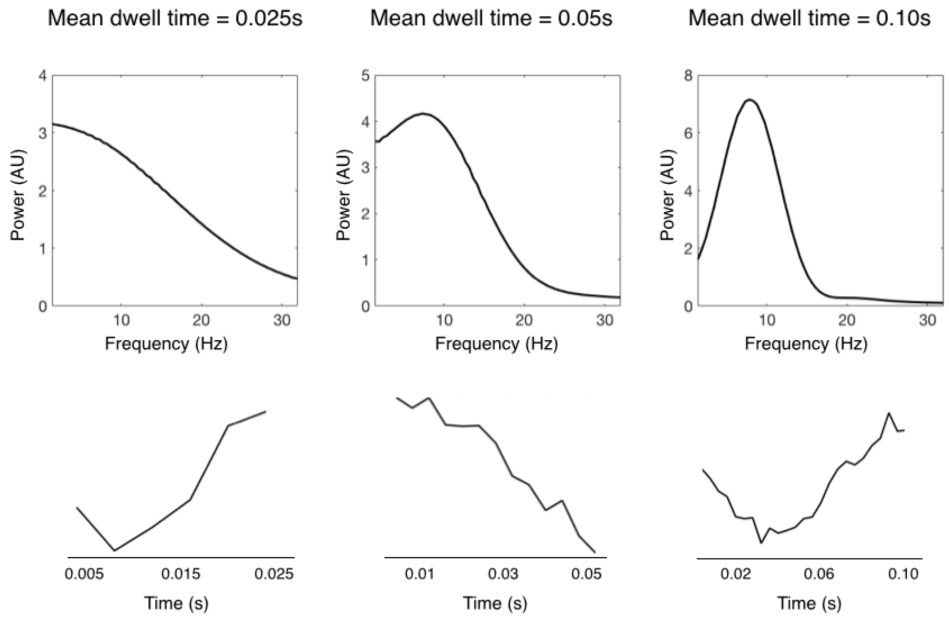

Supplementary Figure 9. **Spectral information from a collection of signal segments extracted from a canonical theta oscillation (8Hz), where the segments are shorter than the theta period (0.125s)**. The length of the segments has either mean 0.025s (left), or 0.05s (middle), or 0.10s (right); examples of the segments are shown in the bottom for each case. Despite the brevity of the segments, the spectral information of the underlying theta wave is correctly calculated.

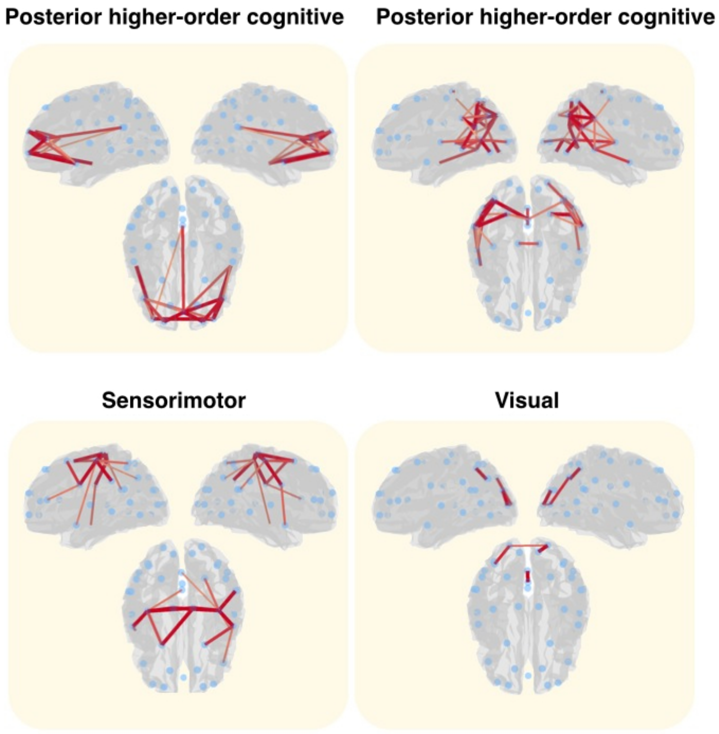

Supplementary Figure 10. **Using an alternative leakage correction method does not affect the results significantly**. Phase-locking connectivity for the two higher-order cognitive (anterior and posterior) states, and the visual and motor states, when the method for leakage correction presented in [78] is applied instead of the method used in this work[25]. The differences between the two methods are not large, with slightly fewer connections for the method introduced in [78].

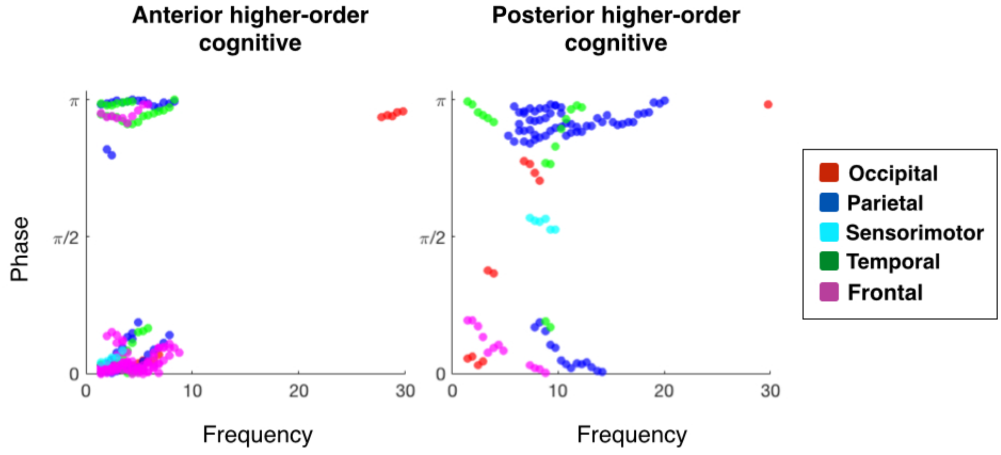

Supplementary Figure 11. **Phase differences between the PCC and the rest of the brain are nearly in-phase (or anti-phase)**. Phase differences between the PCC and the rest of the brain in the anterior and posterior higher-order cognitive states, for those regions that exceed a threshold of 0.05. Different colours indicate different large-scale areas of the brain, as indicated in the legend. Each dot represents the connection of one region to the PCC at a certain frequency.

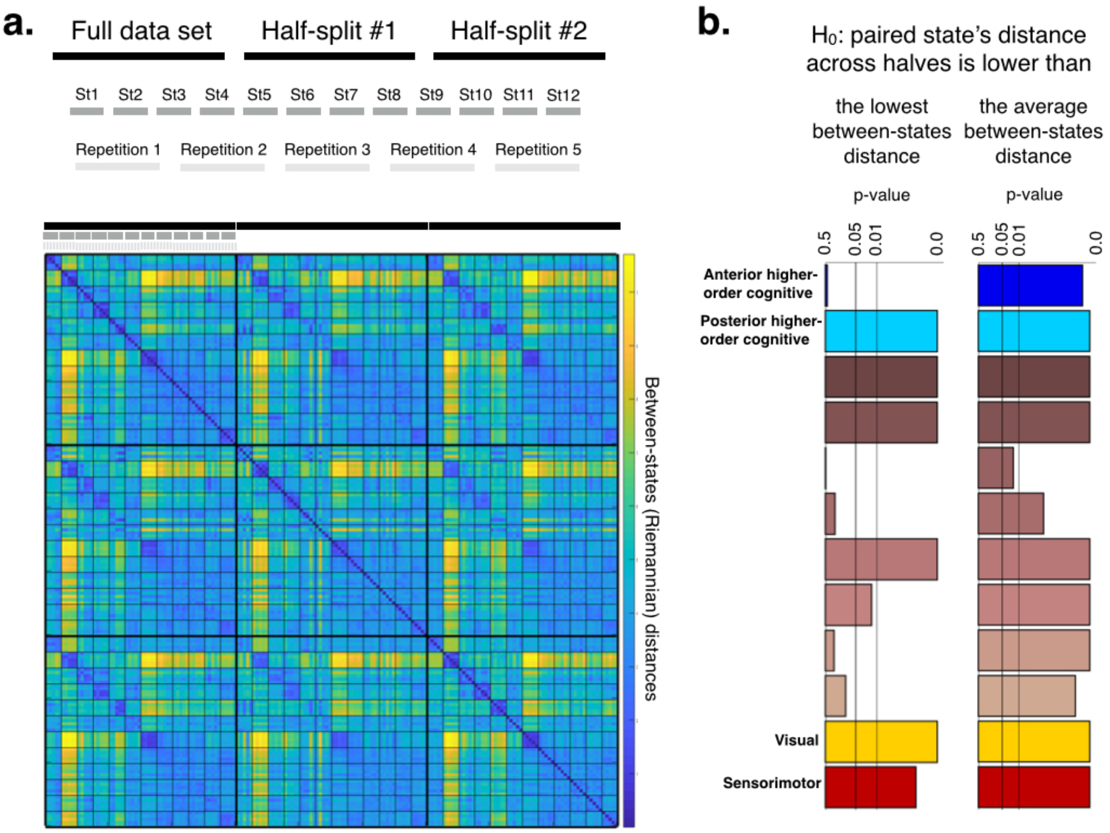

Supplementary Figure 12. **The HMM states are largely reproducible across different runs of the inference algorithm and across half-splits of the data**. (**a**) Riemannian distances between each pair of states, across runs and half-splits. (**b**) Statistical testing on the reproducibility of the states between half-splits. Two tests are implemented: whether the distance between the half-split versions of the same state is lower than that of any pair of states, and whether distance between the half-split versions of the same state are lower than the average between-states distance.

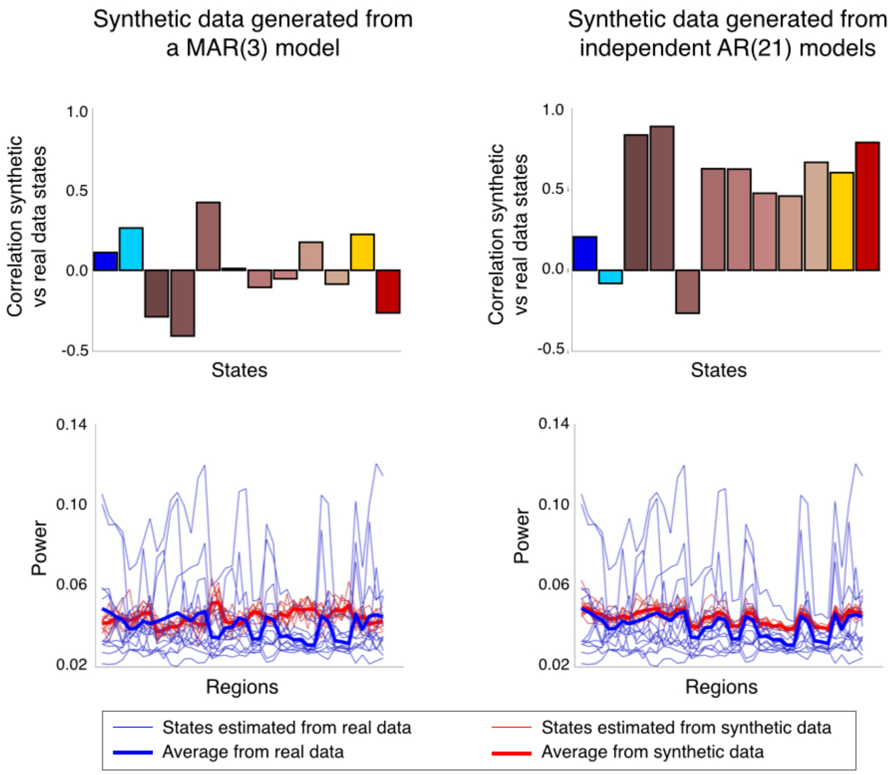

Supplementary Figure 13. **HMM states obtained from synthetic data hold substantial differences with the real-data states**. This is shown for two synthetic data generation models (see **Supplementary Discussion**). On top, correlation between the activation maps between the synthetic and the real maps. Underneath, a depiction of the power values for the real data and the synthetic data states.

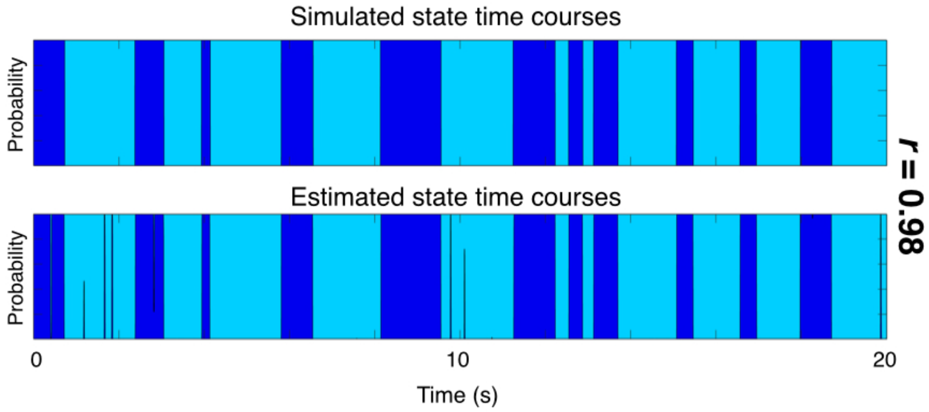

Supplementary Figure 14. **The HMM inference can accurately recover the state time courses of simulated data using Gaussian processes** (see **Supplemental Discussion** for details).

Supplementary References

1. Cole, M.W., Yang, G.J., Murray J.D., Repovš, G. & Anticevic, A. Functional connectivity change as shared signals dynamics. *Journal of Neuroscience Methods* **259**, 22-39 (2016).
2. Duff, E., Makin, T., Smith, S.M. & Woolrich, M.W. (2017). Disambiguating brain functional connectivity. http://doi.org/10.1101/103002.
3. Lowet, E., Roberts, M.J., Bonizzi. P., Karel. J. & De Weerd, P. (2016). Quantifying neural oscillatory synchronization: A comparison between spectral coherence and phase-locking value approaches. PLoS ONE 11: e0146443.
4. Gardner, T.J. & Magnasco M.O. Sparse time-frequency representations. *Proceedings of the National Academy of Sciences of the USA* **103,** 6094-6099 (2006)**.**
5. Huang, N.E., Wu, Z., Long, S.R., Arnold, K.C., Chen, X. & Blank, K. On instantaneous frequency. *Advances in adaptive data analysis* **1**, 177-229 (2009).
6. Bishop, C.M.. Pattern Recognition and Machine Learning (Springer, New York, New York, USA, 2006).
7. Beal, M.J., Ghahramani, Z. & Rasmussen, C.E. The Infinite Hidden Markov Model. *Neural Information Processing Letters* **14**, 577-584 (2002).
8. Vidaurre, D., Abeysuriya, R., Becker, R., Quinn, A.J., Alfaro-Almagro, F., Smith, S.M. & Woolrich, M.W. Discovering dynamic brain networks from big data in rest & task. *NeuroImage*. In press (2017).
9. Vidaurre, D., Quinn, A. J., Baker, A. P., Dupret, D., Tejero-Cantero, A., & Woolrich, M.W. Spectrally resolved fast transient brain states in electrophysiological data. *NeuroImage* **126**, 81-95 (2016).
10. Vidaurre, D., Smith, S.M. & Woolrich, M.W. Brain networks dynamics are hierarchical organised in time. *Proceedings of the National Academy of Sciences of the USA* ***114,*** 12827-12832 (2017).
11. Colclough, G.L., Brookes, M.J., Smith, S.M. & Woolrich, M.W. A symmetric multivariate leakage correction for MEG connectomes. *NeuroImage* **117**, 439-448 (2015).
12. Pascual-Marqui, R.D., Biscay, R.J., Bosch-Bayard, J., Faber, P., Kinoshita, T., Kochi, K., Milz, P., Nishida, K. & Yoshimura, M. Innovations orthogonalization: a solution to the major pitfalls of EEG/MEG "leakage correction". *arXiv*:1708.05931. (2017).
13. Fries, P. Rhythms for cognition: Communication through coherence. *Neuron* **7**, 220-235 (2015).
14. Rasmussen, C.E. & Williams, C.K.I. (2006). Gaussian Processes for Machine Learning. The MIT Press.
